# Queuing Theory Based Co-Channel Interference Analysis Approach for High-Density Wireless Local Area Networks

**DOI:** 10.3390/s16091348

**Published:** 2016-08-23

**Authors:** Jie Zhang, Guangjie Han, Yujie Qian

**Affiliations:** College of Internet of Things, Hohai University, Changzhou 213022, China; 20141931@hhu.edu.cn (J.Z.); 20141923@hhu.edu.cn (Y.Q.)

**Keywords:** co-channel interference, queuing theory, wireless local area networks, high-density environment

## Abstract

Increased co-channel interference (CCI) in wireless local area networks (WLANs) is bringing serious resource constraints to today’s high-density wireless environments. CCI in IEEE 802.11-based networks is inevitable due to the nature of the carrier sensing mechanism however can be reduced by resource optimization approaches. That means the CCI analysis is basic, but also crucial for an efficient resource management. In this article, we present a novel CCI analysis approach based on the queuing theory, which considers the randomness of end users’ behavior and the irregularity and complexity of network traffic in high-density WLANs that adopts the M/M/c queuing model for CCI analysis. Most of the CCIs occur when multiple networks overlap and trigger channel contentions; therefore, we use the ratio of signal-overlapped areas to signal coverage as a probabilistic factor to the queuing model to analyze the CCI impacts in highly overlapped WLANs. With the queuing model, we perform simulations to see how the CCI influences the quality of service (QoS) in high-density WLANs.

## 1. Introduction

**Figure 1:**
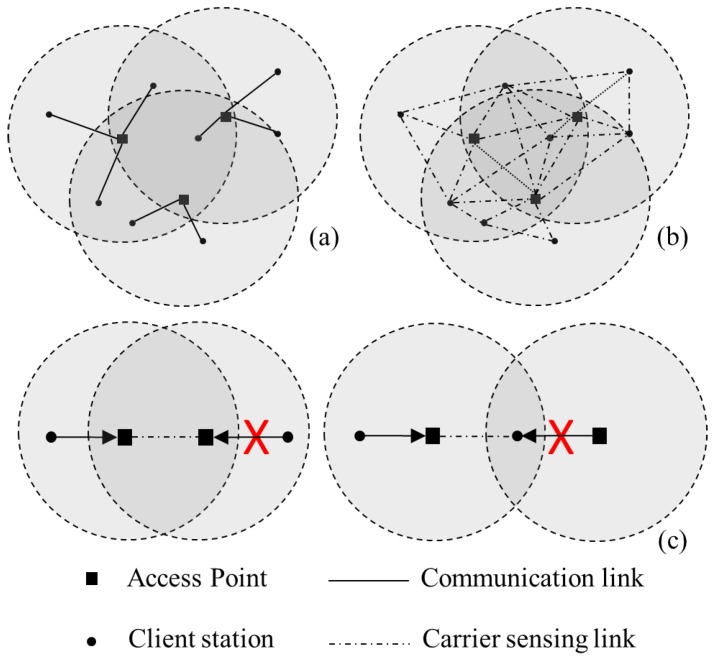
Example of co-channel interference in WLANs: (**a**) data communication topology; (**b**) carrier sensing topology; (**c**) cases of CCI through neighboring AP and neighboring client station.

With the growth of commercial and personal wireless local area networks (WLANs) deployments continuing unabated in recent years, we are witnessing an increasing level of mutual co-channel interference (CCI) among transmissions in public wireless environments, such as in airports, shopping malls, school campuses. The CCI of IEEE 802.11-based networks occurs when multiple networks overlap and trigger the carrier sensing mechanism known as carrier sense multiple access with collision avoidance (CSMA/CA), which is the most widely adapted distributed scheduling protocol in practice. In terms of the “listen before talk” policy of the CSMA/CA, when multiple wireless nodes share the same signal zone, there would be only one node per channel communicating with the access point (AP) at a time. Therefore, in the high-density WLANs combining several overlapped basic station set (BSS) zones, neighboring APs would mutually generate large amounts of CCIs through APs and client stations in the overlapped areas, deteriorating the network quality of service (QoS). Figure 1 illustrates an example of CCIs among overlapped WLANs, where Figure 1a shows a topology of the wireless data networks formed by three BSSs. Figure 1b is the related carrier sensing topology, where all the node pairs with less than four hop distances (see Figure 1c) mutually trigger the CCI in same or overlapped channels. Figure 1c illustrates two instances of CCIs in overlapped areas, when a node pair is communicating and the destination has neighboring nodes in overlapped area, they would become hidden terminals to the source (if they have unreachable distance) which may potentially trigger signal collisions. Hence, CSMA/CA adapts a Request to Send (RTS)/Clear to Send (CTS) handshake protocol to avoid signal collisions from hidden terminals, where RTS for a transmission request and CTS for the response (if the channel is available at the moment), meanwhile any of communications from/to the source and destination’s neighbors would be restricted since they could sense RTS or CTS as well as hidden terminals. However, we can see that the restricted communications would not cause signal collisions and the temporal resources are wasted in case of Figure 1c.

As controlling the network topology and minimizing the overlapped signal zones is a straightforward way of reducing the CCIs in WLANs, many previous researches have focused on bringing effective environmental analysis schemes [1,2]. In IEEE 802.11 WLANs, data can be transmitted successfully even if there are other interfering communications in the same channel, provided the instantaneous transmission power exceeds the joint interference powers by a certain threshold. This threshold factor is known as the signal-to-interference noise ratio (SINR), and is a widely studied environmental analysis factor of CCI management [3,4]. Griffith et al. [5] have presented an integrated PHY and MAC layer model for the IEEE 802.11 MAC, where the PHY layer model computes values for the conditional probability of a frame transmission attempt failure by SINR measurement and the MAC layer model predicts the performance metrics with traffic patterns. SINR-based CCI management generally follows the steps of first collecting the feedback of the signal information from the physical layer. Second, it evaluates the SINR to analyze the channel quality in the application layer. Finally, it manages the wireless topology to optimize the network operations in the MAC layer. Therefore, this usually requires complicated cross-layer architectures [6] difficult to design for complex high-density environments.

In this article, we present a CCI analysis approach that considers the CSMA/CA generated MAC layer packet-queuing-events as CCI factors. In IEEE 802.11-based networks, if the signal propagation range and carrier sensing range are identical, theoretically, there should be no co-channel noise, as every 802.11 channel would adapt the “listen before talk” policy of the CSMA/CA, and only the channel contentions caused by the queuing events would generate the transmission delay and packet dropping. In WLANs, when stations start data transmissions, they will attach a duration field which is known as network allocation vector (NAV) at every MAC frame header to announce the transmission time. The NAV is not only announced by senders but also by receivers through reply messages such as CTS and ACK packets (set to 0), and thus all the neighbors of sender and receiver have to wait for the NAV time to stat other communications. According to such mechanism, if a station’s NAV time for previous communication is not finished but the station has to send another packet, it will store the packet in buffer which will work as a queue. With this assumption in this article, we adapted the queuing theory [7] and mapped the CCI factors to the M/M/c queuing model to analyze complex high-density wireless environment. In our scheme, we assume all the moving events and packet-generating events are randomly occurring, all the nodes in the networks conform to the CSMA/CA protocol, and thus, there is no physical co-channel noise in the environment. The scheme’s flexibility may be weaker than the SINR-based CCI analysis, since it does not take account of geographical interference factors such as physical noise or signal fading. However, the scheme presents an easy adaptable probabilistic analysis of complex channel contention events, bringing a novel CCI analysis approach to high-density wireless environments. Moreover, it does not require any physical layer operations such as SINR measurements, meaning, it is a simpler approach to managing the CCI in practice and is stronger at adapting to complex wireless environments.

The primary contribution of our work is the proposal of the approach of queuing theory based CCI analysis for WLANs which maps the CCI factors to M/M/c queuing factors. And for the secondary contribution, we use a novel CCI analysis factor in our queuing model, which is rate of overlapped signal areas to signal coverage, as the evaluations of signal overlapped area have not commonly studied for CCI analysis in high-density environments because of the high geometrical complexity. The remainder of this article is organized as follows. Section 2 introduces background work. In Section 3, we present the queuing theory based CCI analysis approach, and the following Section 4 is about the M/M/c queuing model and factors our queuing model uses. Section 5 illustrates the simulations of CCI analysis with QoS evaluation. Conclusion follows.

## 2. Related Works

### 2.1. Signal Interference in High-Density Wireless Environment

High-density wireless environment is the outcome of increasing social requirements of wireless services and the rapid growth of wireless devices. Since most of the civilian networks are deployed on-demand by communication services with variable properties, it is very difficult to apply centralized control to these large-scale overlapped wireless areas. Therefore, effective resource utilizations in high-density wireless environments become a focused issue in recent years [8,9], usually with a purpose of saving electrical energy [10,11,12], or improving the network QoS [13,14]. Overlapping networks generate CCI and deteriorate the network QoS, while methods of reducing CCI are studied for reusing the wireless spectrum resources by encoding schemes [15,16], channel assignment schemes [17,18] and reusing the spatial resources by controlling the carrier sensing range [19,20].

Since the WLANs are presently the most popular civilian network (e.g. Wi-Fi), analysis of the CCI from WLANs has been of interest not only for the QoS improvement of the IEEE 802.11-based WLANs themselves [21,22,23], but also for the effective operation or resource optimization of other networks that work in the same spectrum such as the IEEE 802.15.4 wireless personal area networks (WPANs) [24,25]. In general, the CCI of IEEE 802.11-based networks include the PHY layer interference from the noise of the physical medium and the MAC layer interference due to the channel access scheme [26]. In this article, we target the MAC layer CCI generated by carrier sensing from the IEEE 802.11 CSMA/CA protocol. Although the CSMA/CA guarantees certain network reliability [27], it also brings the CCI problem, especially in high-density wireless environments [28,29]. Following works surrounds such interference problems. Ilenia et al. have [26] analyzed the interference effects in IEEE 802.11 based networks, and presented a measurement method with evaluation of packet retransmission and collision rate. Kashyap et al. [30] presented a passive monitoring of wireless traffic with machine-learning approach to understand and estimate the interference in WLANs. Zhang et al. [31] analyzed how network and environmental factors affect the interference oriented MAC protocol, and suggested that design of MAC protocol requires new approaches for dynamic, unpredictable network and environmental settings. Al-Bado et al. [32] pointed that a typical wireless network exhibits time-varying channel conditions, that physical interference models become insufficient for asymmetric interference, which requires better MAC layer models for higher accuracy to measure the interferences. Baid et al. [33] analyzed how the interference among neighboring APs effect network throughput of individual WLANs, and proposed a cooperative client-AP Association scheme which considers both of intra-BSS and inter-BSS carrier sensing topologies to mitigate the interference effects in high-density WLANs. Karimi et al. [34] present a solution for optimization of AP association to mitigate the interference in high-density WLANs, they used centralized mathematical formulation to maximize the AP’s upstream throughput as well as the experience for client stations. Patro et al. [35] proposed a centralized AP management framework for real-world high-density environments, the framework was implemented by two applications, one is for optimizing channel assignments with airtime utilization information from co-located APs and another one is for managing airtime access of neighboring APs to reduce channel contentions as well as mitigate interference.

### 2.2. Queuing Theory Based MAC Analysis

Queuing theory is a mathematical method of analyzing the congestions or delays of waiting in queue; it ranges from the study of simple arrive-service chains to the analysis of arbitrarily complex networks of queues, Markov chain [36] is a well-known example, which are mathematical systems based on the queuing theory, it is usually used to analyze probability distributions in continuous state spaces.

**Figure 2:**
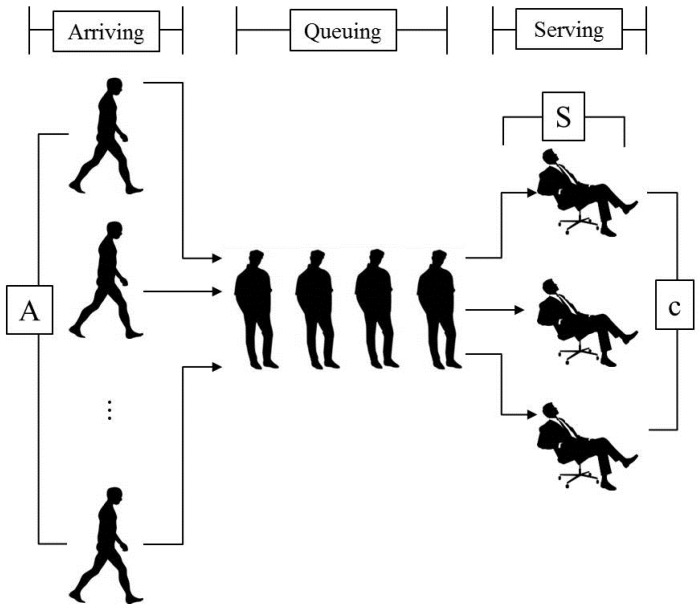
A general queuing model of Kendall’s notation.

In queuing theory, Kendall’s notation is introduced as the standard system used to describe and classify the queuing events. It uses three factors of A/S/c, where A describes the time between arrivals to the queue, S the size of jobs, and c the number of servers at the node. Figure 2 illustrates a basic queuing model with the principles of Kendall’s notation.

In Kendall’s notation, following queuing models are introduced:

- *M/D/1, M/D/c*: M/D/1 is the initial queuing models described in Kendall’s notation, where arrivals are determined by a Poisson process (Also known as Markovian process: M) and service times are deterministic (D) with a service discipline of first-come, first-served. M/D/c is an extension of the M/D/1; it represents the queue length in a system having multiple (c) servers.
- *M/M/1, M/M/c*: Single/multiple servers serve jobs that arrive according to a Poisson process; however the service times follow exponential distribution compare to deterministic service times in M/D/1 and M/D/c.
- *M/G/1, M/G/k*: Extensions of the M/M/1 and M/M/c queue, service times have a general distribution (G). M/G/1 can be used to the cases where different arrivals have different service time distributions, and most metrics for the M/G/k queuing model where k for the number of servers are not known yet and remain an open problem [37].

The principle of the MAC protocols of wireless networks is allocation of packet scheduling. Hence, numerous researches have adapted the queuing theory to diverse wireless environments to measure the MAC or design efficient MAC protocols. Following works introduce recent contributions, which are target IEEE 802.15.4 based sensor networks, IEEE 802.22 based cognitive radio (CR) networks and IEEE 802.11 based mobile ad hoc networks (MANETs). Hairong et al. [38] designed a mathematical model of the MAC access with the queuing theory and then analyzed the superframe structure of the slotted MAC for industrial wireless sensor network (IWSN), the scheme minimized the latency of MAC access while satisfying different packet generating rates with different scale of the network. Azarfar et al. [39] presented a comprehensive modeling and analytical delay analysis for CR networks, they used queuing models with buffering and switching recovery policies to select optimal network parameters, and mentioned that accurate results are difficult to compute due to the problem high dimensionality and complexity. Ozdemir et al. [40] had developed a queuing model to analyze IEEE 802.11 DCF, the model is extended from M/G/1/K where K represents the max queue length and the work targeted at single-hop ad hoc networks. Jia et al. [41] presented an end-to-end delay model with queuing theory to show how the network delay can be analytically determined for given network scenarios, the work targeted at MANETs which are buffer-limited and have uniformly distributed nodes’ locations, and the end-to-end delay is derived by computing its queuing delay and delivery delay.

Recent studies proved that queue-based MAC design to CSMA based networks can achieve high network performance. Li et al. [42] presented a queue analysis based buffer sizing algorithm for WLANs and achieved significant enhance of network performance in testbed-based experiments, which implied that the queue analysis is an efficient approach in MAC design. Lee et al. [43] proposed a new MAC protocol called O-DCF (optimal distributed coordinate function) which modifies the rule of adapting CSMA parameters, O-DCF uses the contention factors to automatically control queuing length at MAC layer without knowing topological knowledge of neighboring interference, and achieves near-optimality in terms of throughput and fairness; after that, Lee et al. [44] proposed a protocol called A-DCF(advanced DCF), which inherits the framework of O-DCF and re-designed for properties of TCP based networks, such as ACK clocking and interference between data frame and ACK frame. Choi et al. [45] modeled the IEEE 802.11 WLANs as closed queuing networks and derive the performance of the CSMA/CA based on the queuing theory, then they proposed a distributed packet scheduling scheme that improves the short-term fairness to the IEEE 802.11 based networks with a runtime estimation scheme that calculates the number of contending stations. Pourmohammad et al. [46] presented a cross-layer analytical model to evaluate QoS parameters such as delay, jitter and throughput in CSMA based networks. The model was developed in queuing theory which well investigates the stochastic behavior of data transmission.

Our work is highly motivated by above contributions. We target at CCI effects in CSMA based WLANs with M/M/c queuing model. Analysis of the CCI depends on the availability of MAC measurements, but this is not always reliable in high-density WLANs because of the complexity of network traffic from overlapped wireless topology. Therefore, in this article, we present a practicable queuing model oriented CCI analysis approach, as a MAC measurement scheme for resource analysis in high-density wireless environments.

## 3. Co-Channel Interference Analysis Based on Queuing Theory

We describe the queuing-theory-based CCI analysis approach in this section. Apparently, the characteristics of the CSMA/CA-based high-density WLANs lead to massive channel contentions and create large packet queuing events, the main reasons for the QoS deterioration such as the increased network delay and packet dropping rates. Therefore, we focus on the queuing factors of the channel contentions for the CCI analysis. We do not consider physical factors such as the propagation delay and the signal noise, as the purpose of our research is MAC layer CCI analysis for high-density WLANs.

**Figure 3:**
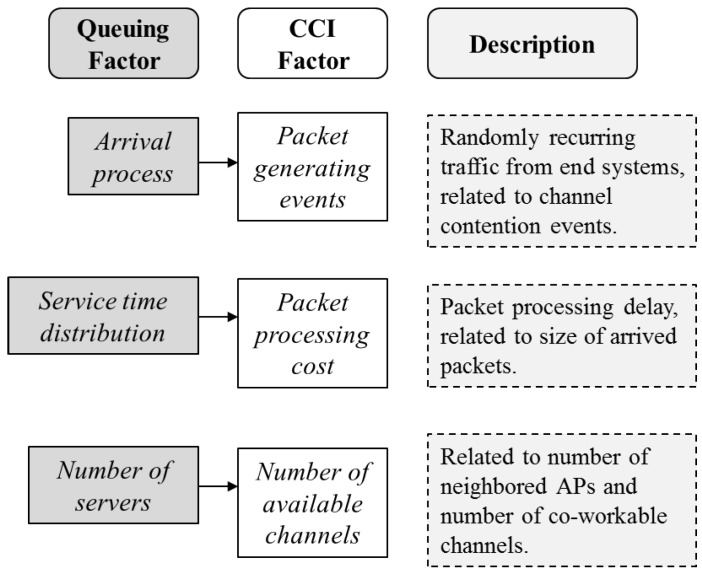
Relationship between queuing model and CCI analysis.

In complex high-density wireless environments, we assume the behaviors of the end users are completely random and map the factors of the CCI analysis in the WLANs to the factors of the M/M/c queue to perform a queuing-theory-based CCI analysis. Figure 3 shows the relationship between the CCI factors and the queuing factors.

According to the queuing theory, we propose the M/M/c queue-based CCI analysis approach with the following conditions:

- The packet generating events in high-density WLANs follow the Poisson process with the event rate *λ*, as the specification of end systems and behaviors of the end users are completely random, qualifying as discrete probability distributions.
- Considering the independency and randomness of generated network traffic and the continuity of channel contention events, the packet processing cost in a given period follows an exponential distribution with average service rate *μ*.
- APs of the WLANs can use *c* non-interfered channels of public band to process the packet generating events. If there are more than *c* packets need to be processed at the same time, the packets queue in a buffer which is of limitless size.

With above conditions, the probability that CCI cause a generated packet to wait in the queuing buffer can be expressed by C(c,λ/μ) which is referred to as Erlang’s C formula as Equation (1) where N is the total traffic offered in units of erlangs.

(1) $$C\left(c,\lambda /\mu \right)=\frac{\frac{N^{c}}{N!}\frac{c}{c-N}}{\sum_{i\;=\;0}^{c-1}\frac{N^{i}}{N!}+\frac{N^{c}}{N!}\frac{c}{c-N}}$$

Then the average number of the packets n in the environment is given by following Equation (2).

(2) $$n=\frac{\frac{\lambda }{c\mu }}{1-\frac{\lambda }{c\mu }}C\left(c,\lambda /\mu \right)+\frac{\lambda }{\mu }$$

And the average processing delay of all generated packets t which includes queuing delay and processing delay is given by following Equation (3).

(3) $$t=\frac{C(c,\lambda /\mu )}{c\mu -\lambda }+\frac{1}{\mu }$$

Finally, the QoS factors such as network throughput, packet delay can be evaluated with the M/M/c queue, average network throughput in every channel can be calculated as Recevedbits/(c×n×t). However, we have to consider that the event rate *λ* increases by the density of wireless stations in WLANs, and thus, QoS analysis in high-density environment requires the information of signal-overlapped zones of BSSs to estimate the number of potential CCI sources in the environment.

**Figure 4:**
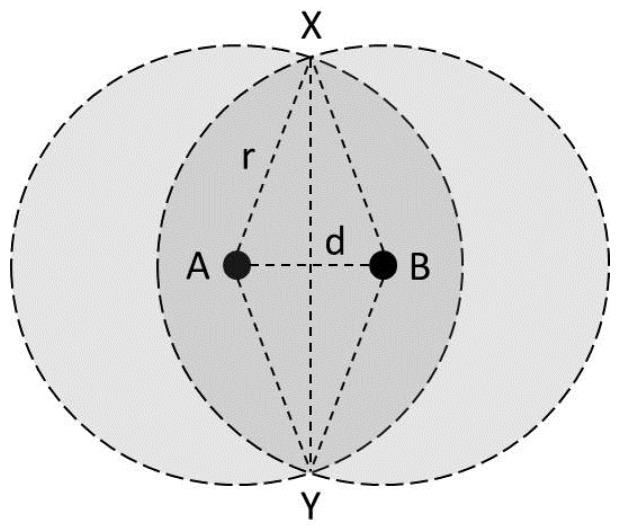
OCR calculation method, overlapped area among two BSSs’ signal coverage can be calculated by measuring the distance between the APs.

In high-density WLANs, most of the CCI occur when the coverage of the multiple BSSs overlap and trigger the channel contentions. Thus, we adopt a signal Overlapped area to signal Coverage Ratio (OCR) as a novel CCI analysis factor to represent a multi-server queuing environment. The OCR is related to the probability of the end users being present in overlapped signal zones and triggering CCI from a neighboring BSS. The OCR is a mathematical factor calculated geometrically from the distance between neighbored APs and the signal range of the local AP, with Figure 4 and Equation (4) showing the OCR calculation method.

(4) $$OCR=\frac{2(\frac{2\arccos(2r/d)}{360}\pi r^{2}-\sqrt{r^{2}-{\left(d/2\right)}^{2}}d)}{\pi r^{2}}$$

According to Figure 4, calculating the overlapped area requires knowing the distance of each node from the APs which can be calculated by Friis equation [47], using measurements of the received signal strength (RSS). The overlapped area is calculated by the following method: calculate the sectorial area of AXY(s), the triangular area of AXY(t), and the overlapped area would be 2×(AXY(s)−AXY(t)).

## 4. M/M/c Queuing Model for CCI Analysis

In this section, we propose the M/M/c queuing model to analyze the effects of CCI in WLANs for further QoS evaluations. We adapt the network parameters of the IEEE 802.11 standard to the queuing model as in Table 1.

According to Table 1, M/M/c parameters are evaluated as in Table 2.

Following pseudo-codes explain the processes of our queuing model. We assume high-density WLANs where APs of neighbored BSSs’ may mutually sense each other and potentially trigger CCI in signal-overlapped zones, if they use the same channel. 

**Pseudo-Code 1:** Queuing process: arriving → queuing
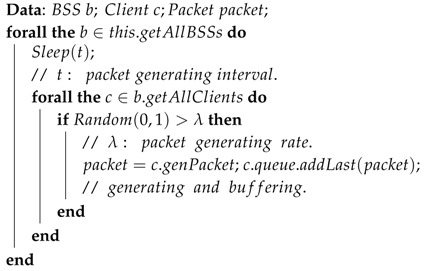


In our queuing model, client stations in each BSS generate random size packets with a given rate in a certain period, and then try to transmit them to local AP through channel contentions with their contention window (CW). According to the DCF of the IEEE 802.11 MAC, stations will start transmitting packet only if associated channel of local BSS or neighboring overlapped BSSs is not occupied; however, collisions still happen because two or more stations may sense same available channel and start transmitting packets at the same time. In such cases, the stations have to wait another channel contention period to retransmit the packets and double the CW. Meanwhile, if the stations’ previously generated packets were not processed yet, then newly generated packets have to be buffered by queues. Again, packets in the head of the queuing buffers will start another channel contention with their stations’ CW, when packets are successfully transmitted, CW will be decremented. In our model, we assume the client stations open one session to their APs, and the number of co-workable channels is consider as three, since the commonly adopted 2400–2483.5 MHz WLANs frequency can associate three none-overlapped 22 MHz WLANs channels simultaneously. 

**Pseudo-Code 2:** Queuing process: queuing → serving
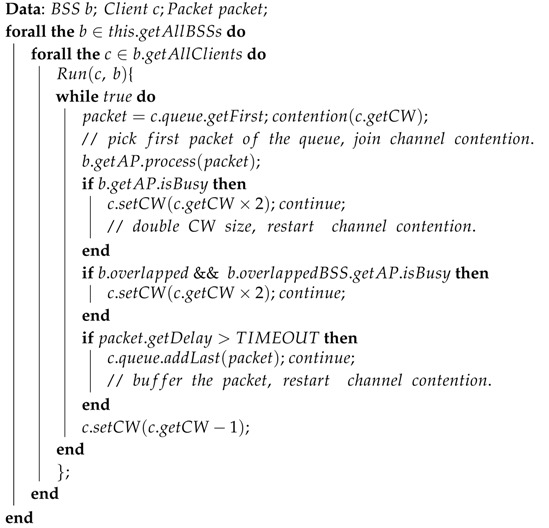


To analyze high-density environments, we adopt the OCR as a probabilistic parameter of the packet arrival events. In case the mean-density of client stations is fixed and a local AP A has *N* clients in its signal zone, an overlapped neighbor AP B would have additional (1−OCR(AB))×N clients in its non-overlapped signal zone. That means the network would generate N+(1−OCR(AB))×N packets in one arrival interval reaching the mutually interfered APs, another overlapped AP C’s additional serviceable clients would be approximately (1−OCR(AC))×(1−OCR(BC))×N, subsequent AP D’s would be (1−OCR(AD))×(1−OCR(BD))×(1−OCR(CD))×N, and so on, the procedures are expressed by Equation (5), where *c* is the number of overlapped APs and *n* is the number of client stations in the wireless zones.

(5) $$n=\sum_{i=1}^{c}N\prod_{j=0}^{i}\left(1-OCR_{ij}\right)$$

The M/M/c model uses the OCR oriented packet generating calculation to estimate the packet arriving events, and the DCF protocol of IEEE 802.11 MAC to emulate the packet queuing events, finally, evaluate QoS through the queuing progresses and analyze the CCI effects in high-density environments.

## 5. Quality of Service Evaluation with Queuing Model

**Figure 5:**
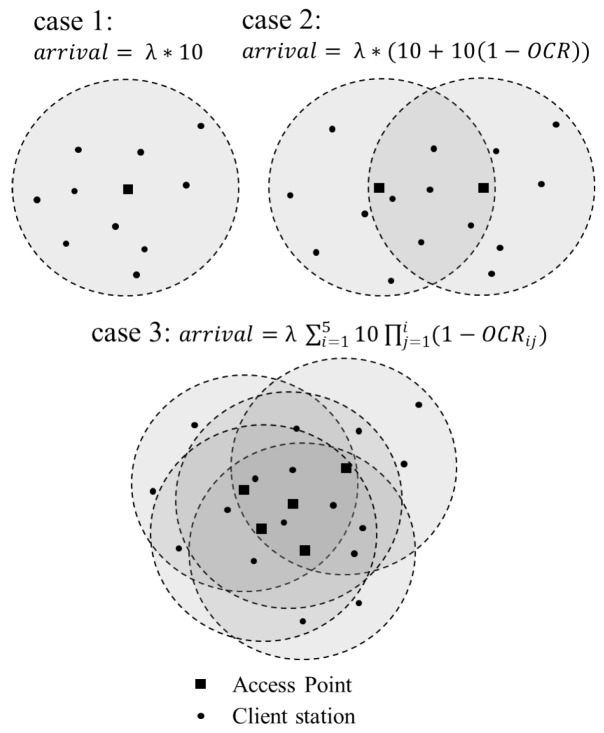
An example of network topology for queuing simulations.

In this section, we perform the M/M/c queuing model oriented network simulations for high-density WLANs. The QoS is evaluated by following parameters in our works (Table 3):

The network topology is organized as follows, client stations are placed at a wireless zone which has mean-density of ten stations per one AP’s signal coverage. We consider only uplink data transmissions, where each client station randomly generates packets and delivers them to the AP placed at reachable range, and thus available channels of each AP work as servers of queuing model and client stations trigger the arrival and queuing events. Figure 5 illustrates examples of our simulation topology, we simulate three scenarios of single AP case, two interfered APs case and five interfered APs case with the M/M/c queuing model. In the simulations, the packet generating events of end systems follow an event rate of 0.3 in every channel contention interval, and the size of generated packet is a random value among the MAC protocol data unit (MPDU, 34–2346 bytes) payload that lead the packet processing costs to exponential distribution.

The simulation program is written in Java language with Eclipse IDE. First, it simulates packet generating events at client side, by generating instances of client stations with pre-defined mean-density and OCR values among APs. Then the program generates a static queue instance which is shared by all of the stations to simulate arriving and queuing events. Finally by the queue instance, it simulates packet processing events at AP side and then calculates the QoS parameters. Functions of our simulation program include packet generation, packet buffering, channel contention, collision capturing, packet retransmission and so on. The simulations are based on following assumptions:

1 Only uplink (client to AP) data transmissions.
2 Each AP uses multi-channel data transmission scheme with three interference-free channels.
3 Packet arrival rate is dynamically changed by channel contention period, means that the client stations generate packet on-demand by the network congestion levels.
4 Queue size is unlimited.
5 Locations of client stations are known (for client-AP associations).
6 Physical noises are not considered.

With such assumptions, if neighboring APs can sense each other, AP-level CCI will be triggered that only one client of both BSSs can access local AP at a time in same channel; and if clients are placed in neighbor AP’s signal-overlapped zone, client-level CCI will be triggered and it would interfere neighbor BSS not to access local AP. The purpose of simulation is to see the effects of CCI on network QoS in high-density environment.

**Figure 6:**
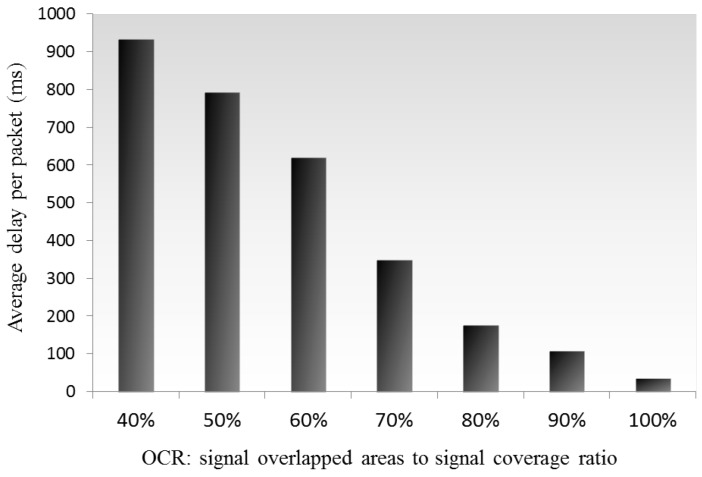
Averaged packet transmission delay in fixed mean-density WLANs with different OCR levels.

With Equation (4), we calculate that if two APs can mutually sense each other the minimum OCR is approximately 40%, showing the maximum CCI effects in the fixed mean-density WLANs environment. Figure 6 shows that a high OCR results in fewer CCI effects when it is higher than 40%, because a high OCR means smaller wireless coverage of neighboring APs, and less CCI sources. In case the OCR is lower than 40%, the distance between the APs that is longer than the carrier sense range, and CCI will not be triggered among the APs—this case will be analyzed later. Following simulations assume a high-density environment formed by mutually reachable APs, and therefore, we set the OCR as greater than 40% to analyze the effects of the CCI on the QoS.

In Ozdemir et al.’s work [40], IEEE 802.11 DCF based queuing simulations showed that when the packet arrival rate reaches a certain value, the delay would rapidly increase because the packet processing speed could not catch up the arriving speed. In [40]’s simulations, the value was 10/s, 20/s, 45/s for 5, 10 and 20 nodes and the delay was increased to ≈600 ms, ≈1 s and ≈2 s respectively, within the values the delay was maintained steady as ≈3 ms. Our simulations shows similar pattern in delay variations, when ten clients share a single AP with an arrival rate of 0.3/contention time (1×Slottime to CWsize×Slottime) and none of neighboring BSSs in the environment, the CCI would only be triggered among those clients and the average delay was maintained at less than 40 ms. This showed an acceptable QoS indication for most communication services. However, for the cases where several APs mutually overlapped, the average delay increased to ≈800 ms with two overlapped APs and to ≈1100 ms with five. In our cases, the arrival rate was not changed but the density of client stations was increased.

**Figure 7:**
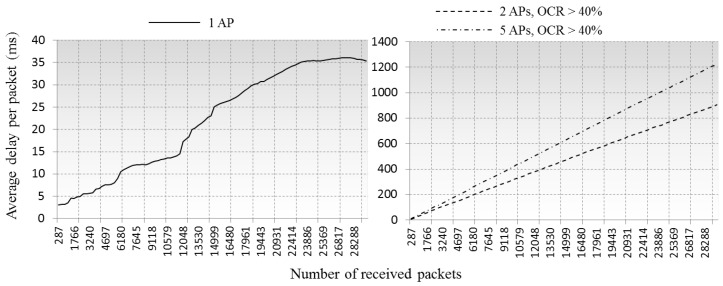
Comparison of averaged transmission delay of arrived packets.

Simulations by Griffith et al. [5] which integrates PHY layer SINR measurement and MAC layer queuing also showed similar patterns as well, which can prove the reliability of our queuing scheme in QoS evaluations even though we did not consider the PHY layer operations. In [5]’s work, the delay was rapidly increased from 3 ms to 10 s with 26 nodes by arrival rate of 0.5/s to 12.5/s at client side and 0.1/s at AP side, and maintained at 3 ms to 5 ms when the arrival rate is under 6/s. Even though they used a quite low arrival rate, but high density of client stations brought very high delays to the networks. For the same reason, the delay in our simulations is increased rapidly in two and five APs cases as Figure 7, because the APs’ serviceability could not satisfy the packet processing requirements from the increased client stations, presenting a hardly acceptable QoS to any of the communication services. This is because if multiple APs can sense each other, no matter how many APs are available for service, only one of them can process the received packets in same channel because of the CCI.

**Figure 8:**
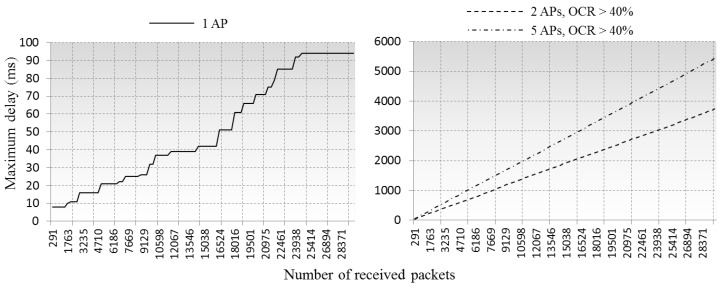
Comparison of maximum queuing delay of arrived packets.

**Figure 9:**
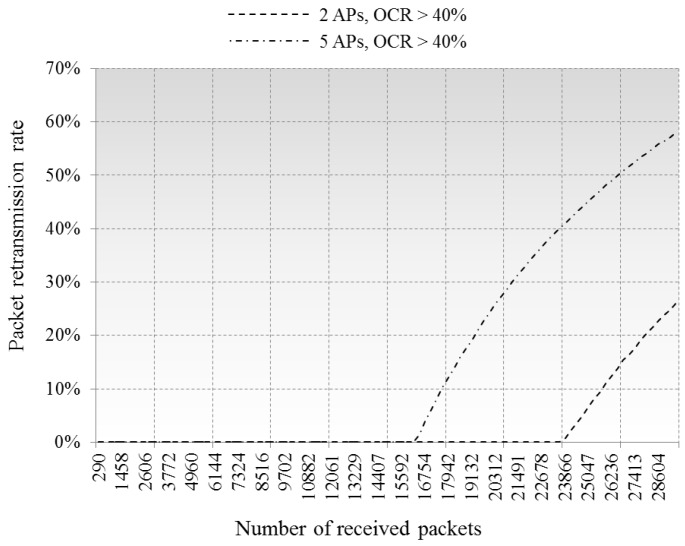
Comparison of racket retransmission rate.

The maximum queuing delay of arrived packets is compared in Figure 8. While ≈30,000 packets arrived at the APs, the longest queuing time was under 100 ms in case of single AP processing the packets from the clients. By contrast, this increased to ≈3500 ms and ≈5500 ms in cases of two and five overlapped APs, respectively. The simulations did not take account of the packet loss events because our queuing model only considers MAC layer CCI, not physical affects. However, packet dropping and retransmission events would occur if the queuing delays are longer than TCP timeout. Figure 9 shows the packet retransmission rate with 3 s timeout, cases of 2 APs and 5 APs are compared, we did not put in the single AP case because it shows near zero packet retransmission rate.

**Figure 10:**
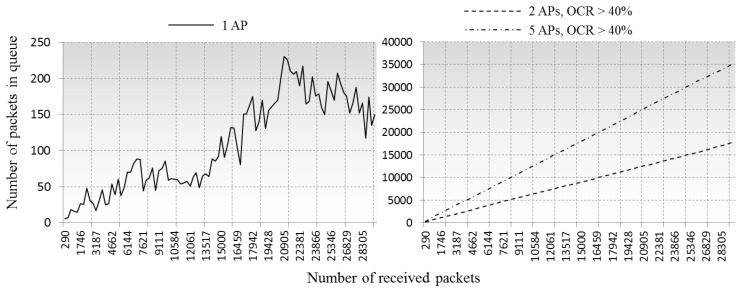
Comparison of number of waiting packets in queue.

**Figure 11:**
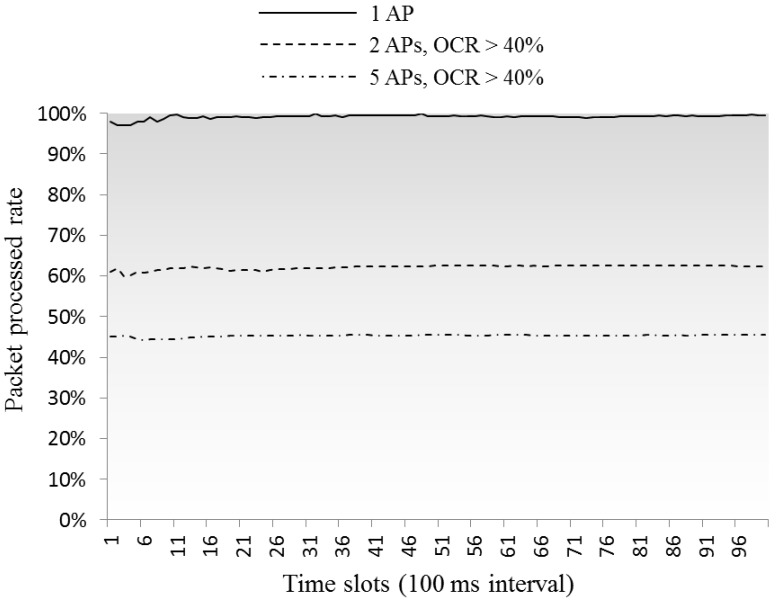
Rate of number of processed packets to number of arrived packets.

**Figure 12:**
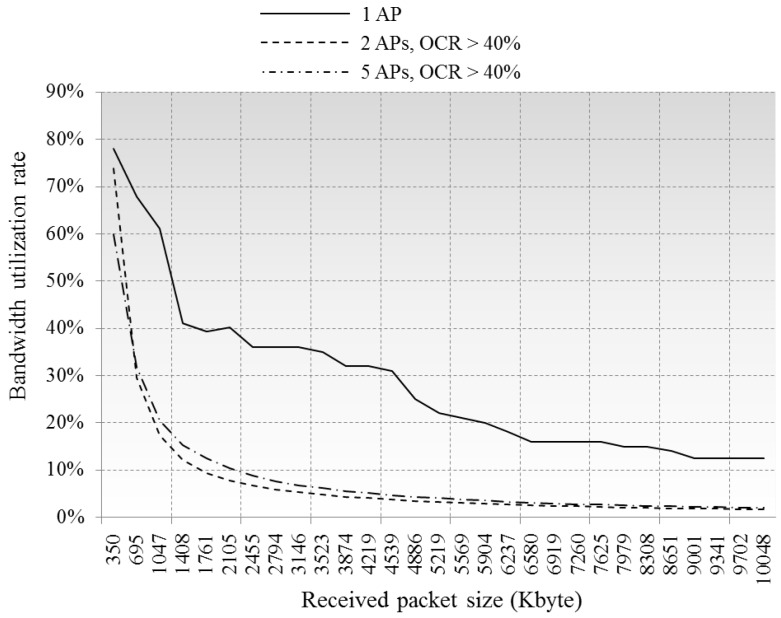
Comparison of bandwidth utilization rate.

Figure 10 illustrates the variations in the number of buffering packets in the queue, and the rates of the processed packets are shown in Figure 11. The results in Figure 11 indicate the AP’s serviceability in different CCI environments, being near 100% in case of a single AP case, and decreasing to ≈60% and ≈45% in cases of two and five overlapped APs, respectively. Figure 12 is about the bandwidth utilization rate in each environment, where the real-time network throughput is calculated as *Received packet size/(Average delay per packet × Number of received packet)*, then the bandwidth utilization rate is calculated as *Network throughput/Maximum data rate* (54 Mbps). From Figure 12, we can imagine how much frequency resources can be used by actual data communications in high-density environment, in our simulations, 12% around in single AP case and less than 3% in 2AP and 5AP cases, the rests are consumed by control traffic and queuing delay.

The simulations shown before assume that the OCR between each AP is higher than 40%, which means distances between the APs are shorter than the APs’ carrier sensing range, and that CCI always exists among the APs. The following simulation shows different cases, where we assume the OCR is lower than 40% and the APs would not directly trigger CCI to others, but indirect CCI among the APs would be triggered through clients placed at overlapping signal zones (see Figure 1c).

**Figure 13:**
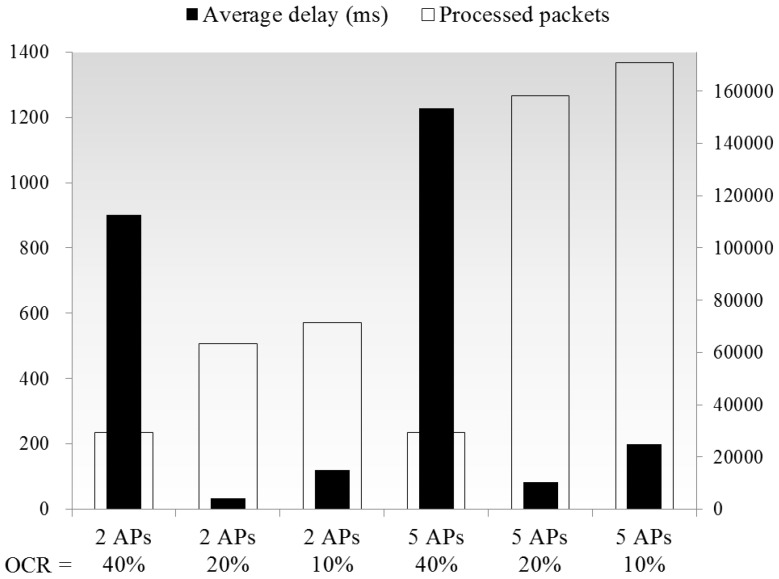
Comparison of averaged delay and number of processed packets in different OCR cases.

Figure 13 shows the QoS in the case of OCRs among APs being less than 40%, and the end to end delay is significantly improved while the OCR reduces from 40% to 20%. Because in case the OCR is lower than 40%, the distances between the APs would be longer than their carrier sensing ranges, and there would be no CCI among the APs. Therefore, multiple APs could utilize the same frequency simultaneously in non-overlapping zones, while the 20% and 10% OCR cases result in similar delays as in the single AP case of Figure 7. And the delays in the 10% OCR cases are higher compared to those for the 20% cases, the reason is same as in the simulation shown in Figure 6, as a lower OCR also means larger wireless coverage with more packet arriving events, and the number of processed packets is improved by the increase of AP numbers, as expected.

## 6. Conclusions

The main reason of the resource constraint in today’s high-density WLANs is increased CCI in highly overlapped signal zones caused by the carrier sensing mechanism of the IEEE 802.11 standards. In this article, we presented a novel CCI analysis approach based on the queuing theory to contribute a practicable QoS evaluation to high-density WLANs. We simulated the CCI in high-density WLANs with an M/M/c queuing model and a new CCI factor introduced as OCR. Some of the simulation results were compared to relevant works and showed that our CCI analysis approach and the queuing model are practicable to QoS evaluations in WLANs, and since our methods target only MAC measurements with probabilistic factors, it is also easily adaptable to complex high-density wireless environments.

## Figures and Tables

**Table 1:** Network parameters in the queuing model.

	Parameter	Value
(1)	MPDU	34–2346 (byte)
(2)	PLCP Preamble	144 (bit)
(3)	PLCP Header	48 (bit)
(4)	Bit per OFDM symbol	216 (bit)
(5)	Symbol processing time	4 (μs)
(6)	Maximum data rate	54 (Mbps)
(7)	ACK frame length	112 (bit)
(8)	SIFS and DIFS	10 + 50 (μs)
(9)	Contention Window (CW)	32–1024
(10)	Slot time	20 (μs)
(11)	Available bandwidth	2400–2483.5 (MHz)
(12)	Bandwidth per channel	22 (MHz)
(13)	Channel gap	5 (MHz)

**Table 2:** M/M/c factors of queuing model.

M/M/c Factor	Value	Description
Arrival interval	Random(1, (9)) * (10) + (8)	Related to event rate and queuing delay
Service time	(Random(1) + (2) + (3) + (7))/(4) * (5)	Related to size of generated packets and transmission rate
Number of servers	(11)/((12) + (13))	Related to available bandwidth and channel properties

**Table 3:** QoS evaluation parameters in queuing model.

	QoS Parameter	Description
(1)	Average delay	Average end to end delay, includes queuing delay and packet processing delay.
(2)	Maximum delay	Maximum end to end delay.
(3)	Packet retransmission rate	Rate of number of processed packets to number of retransmitted packets.
(4)	Queue size	Total number of packets in the queue in given period.
(5)	Processed packets	Total number of processed packets in given period.
(6)	Bandwidth utilization rate	Rate of real-time throughput to maximum data rate.
